# A novel aliasing-free subband information fusion approach for wideband sparse spectral estimation

**DOI:** 10.1186/s13634-017-0494-8

**Published:** 2017-08-23

**Authors:** Ji-An Luo, Xiao-Ping Zhang, Zhi Wang

**Affiliations:** 1 Key Lab for IOT and Information Fusion Technology of Zhejiang, Hangzhou Dianzi University, Hangzhou, 310018 China; 2 Department of Electrical & Computer Engineering, Ryerson University, 350 Victoria Street, Toronto, Ontario, M5B 2K3 Canada; 3 State Key Lab of Industrial Control Technology, Zhejiang University, Hangzhou, 310027 China

## Abstract

Wideband sparse spectral estimation is generally formulated as a multi-dictionary/multi-measurement (MD/MM) problem which can be solved by using group sparsity techniques. In this paper, the MD/MM problem is reformulated as a single sparse indicative vector (SIV) recovery problem at the cost of introducing an additional system error. Thus, the number of unknowns is reduced greatly. We show that the system error can be neglected under certain conditions. We then present a new subband information fusion (SIF) method to estimate the SIV by jointly utilizing all the frequency bins. With orthogonal matching pursuit (OMP) leveraging the binary property of SIV’s components, we develop a SIF-OMP algorithm to reconstruct the SIV. The numerical simulations demonstrate the performance of the proposed method.

## Introduction

Wideband direction-of-arrival (DOA) estimation has been a popular area of research due to the various applications in radar, sonar, seismology, communications, astrophysics, and many other fields [1–3]. Traditional wideband array processing is to decompose the wideband signals into many narrowband signals with a filter bank or the discrete Fourier transform (DFT), and two categories, referred to as incoherent signal subspace method (ISSM) [4] and coherent signal subspace method (CSSM) [5], are utilized to realize wideband DOA estimation. The ISSM estimates the DOAs independently and average them over all the bins. The performance of ISSM may deteriorate with low signal-to-noise ratio (SNR) frequency bins and coherent sources. The CSSM align the signal subspaces by transforming the observation vectors associated with each bin into the focusing subspace and can deal with coherent sources by averaging the subspace-aligned covariance matrices. Compared with ISSM, CSSM can enhance DOA resolution and improve the accuracy of DOA estimates at low SNR. However, CSSM requires an initial DOA estimates, and the precision of DOA pre-estimates greatly influences the accuracy of DOA estimation [6, 7].

Recently, a class of sparse signal representation (SSR) methods provide a new perspective for wideband DOA estimation [8–11]. The DOA estimation problem can be formulated as recovering a spatial sparse signal vector or matrix by minimizing the residual norm under sparsity constraint. Basically, the wideband SSR methods in frequency domain decompose the received signals into narrowband subbands and estimate spatial spectra in each frequency bin. One of the most successive *ℓ*_1_-norm-based SSR algorithms for DOA estimation is *ℓ*_1_-SVD (Singular Value Decomposition) [12], which reduces the computational complexity by SVD. Hyder and Mahata [13] presented a joint *ℓ*_2,0_-norm approximation (JLZA) method and extended it to wideband DOA estimation. Tang et al. [14] showed that the spatial ambiguity can be removed by using multiple dictionaries, each dictionary corresponding to a judiciously chosen frequency. It should be mentioned that the above SSR methods in frequency domain are generally formulated as a multi-dictionary/multi-measurement (MD/MM) joint optimization problem. These techniques usually do not use all the subband information to estimate DOAs with the aim of reducing unknown variables. Similar to ISSM, the performance of joint optimizing a MD/MM problem may deteriorate rapidly if subband signals with low SNR are chosen. Liu et al. [15] proposed a wideband covariance matrix sparse representation (WCMSR) method for DOA estimation. The WCMSR method uses time domain measurements and has its limitation for spatial nonambiguity because the spatial aliasing is frequency dependent.

In this paper, we reformulate the MD/MM problem as a sparse indicative vector (SIV) recovery problem and propose a new subband information fusion (SIF) method to estimate the SIV by jointly integrating all the frequency bins together. Thus, the number of unknown variables can be reduced dramatically. By introducing the SIV, an extra system error is also generated. We show that such error term can be ignored under certain conditions. Compared with the traditional wideband DOA estimation methods, the proposed approach does not rely on focusing technique to average the subspace matrices. By using the binary property of the SIV, we design a binary constrained orthogonal matching pursuit (OMP) algorithm to recover the SIV. The developed algorithm is called SIF-OMP algorithm.

The organization of this paper is as follows. In Section 2, we review the SSR model for wideband DOA estimation. The new SIF method is presented in Section 3. The SIF-OMP algorithm is implemented in Section 4, together with the convergence properties of the SIF-OMP algorithm. Numerical simulations are carried out in Section 5 to demonstrate the performance of our algorithm, and Section 6 gets the conclusion.

## Problem formulation and existing methods

Consider a uniform linear array (ULA) of *N* omnidirectional sensors working together to estimate the spatial location parameters of *Q* wideband sources and *Q* is assumed to be unknown. The sensors of the ULA are equally placed on a line with spacing *d* which is not necessary to be smaller than half a wavelength. Let $\Theta =\left \{\theta _{i}\right \}_{i=1}^{L}$ denote the set of a sampling grid of all possible source locations, *L*≫*N*, *L*≫*Q*. We assume that the grid is fine enough that *Θ* can represent the true source locations, e.g., $\{\theta _{i_{1}},\theta _{i_{2}},\ldots,\theta _{i_{Q}}\}\in \Theta $. The subscript *i*_*q*_, *q*=1,2,…,*Q*, is used to index the position of $\theta _{i_{q}}$.

For each sensor, the time-samples are split into *M* segments, where for each segment, *K* frequency bins are obtained by a bank of narrowband filters or the discrete Fourier transform [7]. Let *s*_*k*,*q*_(*m*) denote the *q*th source signal at frequency *ω*_*k*_ computed for the *m*th segment, *k*=1,2,…,*K* and *m*=1,2,…,*M*. The sparse representation perspective transforms the DOA estimation problem into sparse spectrum recovery problem. We use *v*_*k*,*i*_(*m*) to denote the *k*th frequency coefficient for the *m*th segment corresponding to the *i*-th grid, *i*=1,2,…,*L*. Similarly, we use *y*_*n*,*k*_(*m*) to represent the measurement data at the *n*th sensor for the *m*th segment at frequency *ω*_*k*_. By stacking all measurements into a vector, the output of the array can be expressed as

1 $$ \boldsymbol{y}_{k,m}=\sum_{i=1}^{L}\boldsymbol{a}_{k}(\theta_{i})v_{k,i}(m) + \boldsymbol{w}_{k,m}  $$

where ***y***_*k*,*m*_=[*y*_1,*k*_(*m*),*y*_2,*k*_(*m*),…,*y*_*N*,*k*_(*m*)]^*T*^ is the measurement vector, ***w***_*k*,*m*_ represents the *N*×1 additive noise vector, ***a***_*k*_(*θ*_*i*_) is the steering vector with respect to the *i*-th grid and it can be written as

2 $$ \boldsymbol{a}_{k}(\theta_{i}) = \left[1, e^{-j\omega_{k}\frac{d}{c}\sin\theta_{i}},\ldots,e^{-j\omega_{k}(N-1)\frac{d}{c}\sin\theta_{i}} \right]^{T}  $$

where *c* is the speed of the signal propagation. We now introduce an overcomplete basis matrix ***A***_*k*_=[***a***_*k*_(*θ*_1_),***a***_*k*_(*θ*_2_),…,***a***_*k*_(*θ*_*L*_)]. The sparse representation model in (1) can be expressed concisely

3 $$ \boldsymbol{y}_{k,m}=\boldsymbol{A}_{k}\boldsymbol{v}_{k,m} + \boldsymbol{w}_{k,m}  $$

where ***v***_*k*,*m*_=[*v*_*k*,1_(*m*),*v*_*k*,2_(*m*),…,*v*_*k*,*L*_(*m*)]^*T*^ is called “virtual source" vector and it is the sparse representation of true source vector ***s***_*k*,*m*_= [*s*_*k*,1_(*m*),*s*_*k*,2_(*m*),…,*s*_*k*,*Q*_(*m*)]^*T*^. The nonzero entries of ***v***_*k*,*i*_ represent true sources and zero otherwise. Clearly, $v_{k,i_{q}}(m)=s_{k,q}(m)$. When all *M* segments are available, we define ***Y***_*k*_=[***y***_*k*,1_,***y***_*k*,2_,…,***y***_*k*,*M*_]; then, (3) becomes

4 $$ \boldsymbol{Y}_{k}=\boldsymbol{A}_{k}\boldsymbol{V}_{k} + \boldsymbol{W}_{k}  $$

where ***V***_*k*_ is an *L*×*M* matrix, ***W***_*k*_ is an *N*×*M* matrix, and they are defined similarly as ***Y***_*k*_. If the sources are assumed to be stationary during *M* snapshots, then each column of ***V***_*k*_ shares the same sparsity. As shown in the previous literature (see [12–14, 16] and the references therein), the DOA estimation problem can be solved by reconstructing ***V***_1_,***V***_2_,…,***V***_*K*_ from ***Y***_1_,***Y***_2_,…,***Y***_*K*_ within the scope of the MD/MM problem. We define ***A***=blkdiag(***A***_1_,***A***_2_,…,***A***_*K*_), $\boldsymbol {Y}=\left [\boldsymbol {Y}_{1}^{T},\boldsymbol {Y}_{2}^{T},\ldots,\boldsymbol {Y}_{K}^{T}\right ]^{T}$ and $\boldsymbol {V}=\left [\boldsymbol {V}_{1}^{T},\boldsymbol {V}_{2}^{T},\ldots,\boldsymbol {V}_{K}^{T}\right ]^{T}$, where blkdiag(·) denotes the operation to form a block diagonal matrix. Thus, the sparse representation for the MD/MM problem can be described as

5 $$ \begin{aligned} & \underset{\boldsymbol{V}}{\text{minimize}} & & \left\|\boldsymbol{V}\right\|_{0}, & \text{s.t.} & & \|\boldsymbol{Y}-\boldsymbol{A}\boldsymbol{V}\|^{2}\leq\varepsilon, \end{aligned}  $$

where the *ℓ*_0_ norm ∥***V***∥_0_ denotes the number of non-zero rows of a matrix ***V*** and *ε* is an upper bound of the Frobenius norm of the residual error. However, finding such a combinatorial problem requires an enumerative search and is NP hard. Consequently, ∥***V***∥_0_ is replaced by ∥***V***∥_*p*,*t*_, for 0<*p*≤1, *t*≥1, given in [16]

6 $$\begin{array}{*{20}l}  \|\boldsymbol{V}\|_{p,t} = \left[\sum_{i=1}^{L} \left(\|\boldsymbol{\bar{v}}_{i}\|_{t}\right)^{p}\right]^{1/p}, \quad \|\boldsymbol{\bar{v}}_{i}\|_{t} = \left[\sum_{m=1}^{M}|v_{i,m}|^{t}\right]^{1/t} \end{array} $$

where $\boldsymbol {\bar {v}}_{i}\in \mathbb {C}^{1\times M}$ is the *i*th row vector of matrix ***V*** and *v*_*i*,*m*_ is the *m*-th element in the row vector $\boldsymbol {\bar {v}}_{i}$. Taking the *ℓ*_1,2_ mixed norm minimization as an example, the matrix ***V***_*k*_ can be estimated by the following constrained optimization problem [12]:

7 $$ \begin{aligned} & \underset{\boldsymbol{V}}{\text{minimize}} & & \sum_{i=1}^{L} \left\|\boldsymbol{\bar{v}}_{i}\right\| & \text{s.t.} & & \|\boldsymbol{Y}-\boldsymbol{A}\boldsymbol{V}\|\leq\varepsilon. \end{aligned}  $$

The above optimization problem can be solved with standard optimization software, i.e., second-order cone programming (SOCP). However, the optimization procedure should be repeated by *K* times by utilizing all frequency bins *ω*_1_,*ω*_2_,…,*ω*_*K*_, and *K* could be rather large in wideband signal processing. Indeed, it is not efficient to compute ***V*** using (7) for all frequency components. In this paper, instead of estimating matrix ***V***, we solve the DOA estimation problem efficiently by recovering a SIV which is used to represent the location of sources.

## Subband information fusion method

From the sparse representation model in (4), we observe that the matrices ***V***_1_,***V***_2_,…,***V***_*K*_ share the identical sparse structure, where ***V***_*k*_=[***v***_*k*,1_,***v***_*k*,2_,…,***v***_*k*,*M*_], *k*=1,2,…,*K*. The nonzero rows of matrix ***V*** indicate the source locations. To solve the DOA estimation problem, we need to decide which row of the source matrix ***V*** is non-zero from the measurements. Once this is done, say that the *i*th row of ***V*** is non-zero, we can infer that *θ*_*i*_ is one of DOA estimates for the corresponding source. Indeed, it is not necessary to estimate the whole signal matrix ***V*** to get the solution of a direction estimation problem. Actually, the DOA estimation problem can be formulated as recovering a SIV using a SIF method and the estimating of entire matrix ***V*** can be avoided.

### Method

To develop the SIF algorithm, first, we introduce a projection matrix ***C***_*k*,*i*_ for the *i*th spatial grid at frequency *ω*_*k*_ given by

8 $$ {\begin{aligned} \boldsymbol{C}_{k,i} &= \boldsymbol{a}_{k}(\theta_{i})\left[\boldsymbol{a}_{k}^{H}(\theta_{i})\boldsymbol{a}_{k}(\theta_{i})\right]^{-1}\boldsymbol{a}_{k}^{H}(\theta_{i}) \\ &= \frac{1}{N} \left[ \begin{array}{cccc} 1 & e^{j\omega_{k} \frac{d}{c}\sin\theta_{i}} & \cdots & e^{j\omega_{k} (N-1)\frac{d}{c}\sin\theta_{i}} \\ e^{-j\omega_{k} \frac{d}{c}\sin\theta_{i}} & 1 & \cdots & e^{j\omega_{k} (N-2)\frac{d}{c}\sin\theta_{i}} \\ \vdots & \vdots & \ddots & \vdots \\ e^{-j\omega_{k} (N-1)\frac{d}{c}\sin\theta_{i}} & e^{-j\omega_{k} (N-2)\frac{d}{c}\sin\theta_{i}} & \cdots & 1 \\ \end{array} \right]. \end{aligned}}  $$

Let ***ψ***_*k*,*i*_(*m*)=***C***_*k*,*i*_ ***y***_*k*,*m*_ denote the projection of ***y***_*k*,*m*_ onto the range of ***a***_*k*_(*θ*_*i*_). ***Ψ***_*k*,*m*_ denotes an overcomplete dictionary whose *i*th column is ***ψ***_*k*,*i*_(*m*), and it has the following expression

9 $$\begin{array}{*{20}l} \boldsymbol\Psi_{k,m} = \left[\boldsymbol\psi_{k,1}(m), \boldsymbol\psi_{k,2}(m), \ldots, \boldsymbol\psi_{k,L}(m)\right] \end{array} $$

For noiseless case, we get $\boldsymbol {y}_{k,m} = \sum _{q=1}^{Q}\boldsymbol {C}_{k,i_{q}}\boldsymbol {a}_{k}(\theta _{i_{q}}) s_{k,q}(m)$. Inspired by this relationship, we introduce a new *L*×1 binary SIV ***g***=[*g*_1_,*g*_2_,…,*g*_*L*_]^*T*^ whose nonzero elements indicate the location of sources, i.e., ***g*** has the same sparsity structure of source matrix ***V***. As such, the model (4) can be rewritten as

10 $$\begin{array}{*{20}l} \boldsymbol{Y}_{k}&=\left[\sum_{i=1}^{L}\boldsymbol{a}_{k}(\theta_{i})v_{k,i}(1)g_{i},\sum_{i=1}^{L}\boldsymbol{a}_{k}(\theta_{i})v_{k,i}(2)g_{i}\ldots,\right.\\ &\left.\qquad\sum_{i=1}^{L}\boldsymbol{a}_{k}(\theta_{i})v_{k,i}(M)g_{i} \right]+\boldsymbol{W}_{k}  \\ &= \left[\boldsymbol{\Psi}_{k,1}\boldsymbol{g},\boldsymbol{\Psi}_{k,2}\boldsymbol{g},\ldots,\boldsymbol{\Psi}_{k,M}\boldsymbol{g}\right] +\boldsymbol{E}_{k}+\boldsymbol{\bar{W}}_{k} \end{array} $$

where ***E***_*k*_=[***e***_*k*,1_,***e***_*k*,2_,…,***e***_*k*,*M*_] is an *N*×*M* error matrix and the *m*th column of ***E***_*k*_ is given by

11 $$ \boldsymbol{e}_{k,m} = -\sum_{j=1}^{Q}\sum_{q=1,q\neq j}^{Q}\boldsymbol{C}_{k,i_{j}}\boldsymbol{a}_{k}(\theta_{i_{q}})s_{k,q}(m)  $$

$\boldsymbol {\bar {W}}_{k}=[\boldsymbol {\bar {w}}_{k,1},\boldsymbol {\bar {w}}_{k,2},\ldots,\boldsymbol {\bar {w}}_{k,M}]$ is the error matrix and $\boldsymbol {\bar {w}}_{k,m}=\boldsymbol {w}_{k,m}-\sum _{q=1}^{Q}\boldsymbol {C}_{k,i_{q}}\boldsymbol {w}_{k,m}$. In (10), we observe that ***g*** captures the sparsity property of ***V*** in the dictionary ***Ψ***_*k*,*m*_.

Using (10), we can combine all subband information to estimate a single SIV. Let ***X***_*k*_(***g***)=[***Ψ***_*k*,1_ ***g***,***Ψ***_*k*,2_ ***g***,…,***Ψ***_*k*,*M*_ ***g***] denote the sparse representation matrix of ***Y***_*k*_. When all subbands are considered, the new observation model is given as follows

12 $$\begin{array}{*{20}l} \boldsymbol{Y} = \boldsymbol{X}(\boldsymbol{g}) + \boldsymbol{E} + \boldsymbol{\bar{W}} \end{array} $$

where $\boldsymbol {X}(\boldsymbol {g}) = \left [\boldsymbol {X}_{1}^{T}(\boldsymbol {g}),\boldsymbol {X}_{2}^{T}(\boldsymbol {g}),\ldots,\boldsymbol {X}_{K}^{T}(\boldsymbol {g})\right ]^{T}$, ***Y***, ***E*** and $\boldsymbol {\bar {W}}$ are defined similarly as ***V***. Based on the new observation model (12), the following constrained optimization problem can be used for wideband DOA estimation,

13 $$ \underset{\boldsymbol{g}}{\min} \|\boldsymbol{g}\|_{0}, \quad \text{subject to } \|\boldsymbol{Y}-\boldsymbol{X}(\boldsymbol{g})\|^{2}\leq \eta.  $$

where *η* is the upper bound of the Frobenius norm of the residual error with respect to (12). Note that (13) generally subjects to great error bound compared with (5) for each frequency since additional error ***E*** is introduced in the measurement model (12). Note that ***g*** is the only unknown parameter appeared in (13). Thus, all the subband information can be utilized jointly to estimate ***g***. We call it subband information fusion (SIF) method. The SIF method in (13) attempts to find a sufficiently sparse ***g*** in a manner that ***X***(***g***) consistently fits ***Y*** as sparsely as possible. Instead of estimating ***V***_1_,***V***_2_,…,***V***_*K*_ by the MD/MM method stated in (5), the SIF approach only estimates a SIV ***g*** and therefore significantly reduces the number of unknown variables during the estimation process because the number of frequency bins *K* could be rather large in real applications.

### Analysis

In Section 3, the system error ***E*** appeared in (12) is discarded when we solve the optimization problem (13). This leads to loss of information since ***E*** contains the DOA information. This section mainly analyzes the error term ***E*** and addresses the problem when ***E*** can be neglected.

#### Single source case

It is straightforward to rewrite the measurement ***y***_*k*,*m*_ when only a single DOA is required to be estimated,

14 $$\begin{array}{*{20}l} \boldsymbol{y}_{k,m} = \boldsymbol{a}_{k}(\theta)s_{k}(m) + \boldsymbol{w}_{k,m}. \end{array} $$

In consideration of spatial sparsity, ***y***_*k*,*m*_ can also be written as

15 $$\begin{array}{*{20}l} \boldsymbol{y}_{k,m} = \boldsymbol\Psi_{k,m}\boldsymbol{g} + \boldsymbol{\bar{w}}_{k,m}. \end{array} $$

Combining all these measurements together, we obtain

16 $$\begin{array}{*{20}l} \boldsymbol{Y} = \boldsymbol{X}(\boldsymbol{g}) + \boldsymbol{\bar{W}}. \end{array} $$

From (16), we observe that the system error ***E*** does not exist for single source case. Therefore, there is no information loss for single DOA estimation when we formulate the MD/MM problem as a SIV recovery issue.

#### Multi-source case

Let *μ* denote the parameter controlling the value of ***e***_*k*,*m*_ and *μ* is given by

17 $$\begin{array}{*{20}l} \mu_{j,q} &= \frac{1}{N}\boldsymbol{a}_{k}^{H}(\theta_{i_{j}})\boldsymbol{a}_{k}(\theta_{i_{q}}) \\ &= \frac{1}{N}\sum_{n=0}^{N-1}\exp\left(\frac{-j\omega_{k}nd\kappa(\theta)}{c}\right) \end{array} $$

where $\kappa (\theta)=\sin (\theta _{i_{q}})-\sin (\theta _{i_{j}})$. Thus, ***e***_*k*,*m*_ can be written as

18 $$\begin{array}{*{20}l} \boldsymbol{e}_{k,m} = -\sum_{j=1}^{Q}\sum_{q=1,q\neq j}^{Q}\mu_{j,q}\boldsymbol{a}_{k}(\theta_{i_{j}})s_{k,q}(m) \end{array} $$

Note from (17) and (18) that the value of ***e***_*k*,*m*_ is related to *μ*_*j*,*q*_. We need to thoroughly discuss the property of *μ*_*j*,*q*_. According to (17), the magnitude of *μ*_*j*,*q*_ is given by

19 $$\begin{array}{*{20}l} |\mu_{j,q}| = \frac{1}{N}\left|\frac{1-\exp\left(\frac{-j\omega_{k}Nd\kappa(\theta)}{c}\right)}{1-\exp\left(\frac{-j\omega_{k}d\kappa(\theta)}{c}\right)}\right| \end{array} $$

When $\theta _{i_{q}}$ approaches close to $\theta _{i_{j}}$ infinitely, we obtain

20 $$\begin{array}{*{20}l} {\lim}_{\theta_{i_{q}}\rightarrow\theta_{i_{j}}}\kappa(\theta)=0 \quad \text{and} \quad {\lim}_{\theta_{i_{q}}\rightarrow\theta_{i_{j}}}|\mu_{j,q}|=1 \end{array} $$

The above limit conditions show that the error term ***e***_*k*,*m*_ can not be neglected if the angles of any two incident sources are close enough. However, ***e***_*k*,*m*_ can be very small if *κ*(*θ*) is larger than a certain value which can be determined by an upper bound of |*μ*_*j*,*q*_|, namely *B*_|*μ*|_. *B*_|*μ*|_ is given by

21 $$\begin{array}{*{20}l} B_{|\mu|} = \frac{2}{N\left|1-\exp\left(\frac{-j\omega_{k}d\kappa(\theta)}{c}\right)\right|} \end{array} $$

**Fig. 1:**
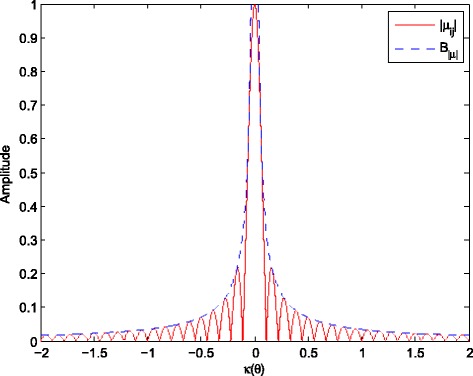
The amplitudes of |*μ*_*j*,*q*_| and *B*_|*μ*|_ with *k*=10. The amplitudes of |*μ*_*j*,*q*_| and *B*_|*μ*|_, *ω*_10_=1.08*π*×10^7^ rad/s, *d*=*π* *c*/(*ω*_0_+*B* *f*/2), *B* *f*=0.2*ω*_0_, *ω*_0_=7*π*×10^7^ rad/s, *c*=3×10^8^ m/s, *N*=7

**Fig. 2:**
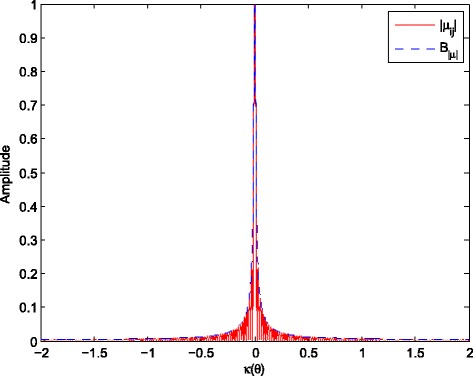
The amplitudes of |*μ*_*j*,*q*_| and *B*_|*μ*|_ with *k*=50. The amplitudes of |*μ*_*j*,*q*_| and *B*_|*μ*|_, *ω*_50_=5.9*π*×10^7^ rad/s, *d*=*π* *c*/(*ω*_0_+*B* *f*/2), *B* *f*=0.2*ω*_0_, *ω*_0_=7*π*×10^7^ rad/s, *c*=3×10^8^ m/s, *N*=7

Figure 1 plots |*μ*_*j*,*q*_| and its upper bound *B*_|*μ*|_ against *κ*(*θ*). Beyond the main lobe, *B*_|*μ*|_ decreases sharply. Hence, if *κ*(*θ*) satisfies

22 $$\begin{array}{*{20}l} \kappa(\theta)\geq\frac{2\pi c}{\omega_{k}Nd} \end{array} $$

then $|\mu _{j,q}|\leq B_{|\mu |}=\frac {2}{N|1-\exp (-j2\pi /N)|}$. This means a small value of ***e***_*k*,*m*_ can be guaranteed when *κ*(*θ*) is larger than a certain threshold. Relatively large values of *ω*_*k*_, *N* and *d* make the threshold at extremely low level; see Fig. 2.

## SIF algorithm

The SIF method proposed in the previous section suggests that we can solve the wideband DOA estimation problem via single measurement vector reconstruction, as long as the system error ***E*** can be neglected. In this section, we first derive an equivalent form for SIF optimization; then, we develop a SIF algorithm to recover the SIV efficiently.

We now develop an equivalent form of (13). Let $\boldsymbol {D}\in \mathbb {C}^{L\times L}$ represent a new dictionary which satisfies ***D*** ^*H*^ ***D***=***Φ***, where

23 $$ \boldsymbol\Phi = \sum_{k=1}^{K}\sum_{m=1}^{M}\boldsymbol\Psi_{k,m}^{H}\boldsymbol\Psi_{k,m}.  $$

One realization of ***D*** is given by the eigenvalue decomposition (EVD) of the Hermitian matrix ***Φ***. Assume that ***Φ*** can be written as ***Φ***=***U*** ***Λ*** ***U*** ^*H*^, where ***U*** is a unitary matrix whose columns are composed by *L* orthonormal eigenvectors and ***Λ*** is a diagonal matrix of eigenvalues. Thus, the dictionary ***D*** has the expression $\boldsymbol {D}=\boldsymbol {U}\boldsymbol {\Lambda }^{\frac {1}{2}}$. Let *t* denote the rank of ***Φ***. We have rank(***D***)=rank(***Φ***)=*t*. We now define a new measurement vector $\boldsymbol \xi \in \mathbb {C}^{L\times 1}$ given such that

24 $$ \boldsymbol{D}^{H}\boldsymbol\xi=\boldsymbol{h}  $$

where $\boldsymbol {h} = \sum _{k=1}^{K}\sum _{m=1}^{M}\boldsymbol \Psi _{k,m}^{H}\boldsymbol {y}_{k,m}$. From the definition in (24), we see that Eq. (24) may be underdetermined because rank(***D*** ^*H*^)=*t*≤*L*. Simultaneously, from

25 $$ \begin{aligned} \textit{rank}(\boldsymbol{\Phi})&=\textit{rank}\left(\left[\boldsymbol{\Phi},\boldsymbol{h}\right]\right) \quad \Longrightarrow \quad \textit{rank}(\boldsymbol{D}^{H})\\&=\textit{rank}\left(\left[\boldsymbol{D}^{H},\boldsymbol{h}\right]\right), \end{aligned}  $$

we can conclude that the solution of (24) exists and ***ξ***=(***D*** ***D*** ^*H*^)^−1^ ***D*** ***h***.

Based on the above definitions, the equivalent expression of (13) is presented as follows:

26 $$ \underset{\boldsymbol{g}}{\min} \|\boldsymbol{g}\|_{0}, \quad \text{subject to } \left\|\boldsymbol{\xi}- \boldsymbol{D}\boldsymbol{g}\right\|^{2}\leq \eta.  $$

Since the SIV ***g*** has the same sparsity structure of ***V***, the following theorem shows that the problem of estimating ***g*** by optimizing (26) is equivalent to estimating ***g*** by using (13).

### **Theorem 1**

Given the optimization problems (13) and (26), the problem of recovering ***g*** through (13) is equivalent to estimating ***g*** by (26).

### *Proof*

See Appendix. □

Theorem 1 suggests that the wideband DOA estimation problem can be solved by optimizing (26) using existing methods, e.g., OMP. We consider the OMP for the recovery of ***g*** based on the measurement vector ***ξ***. OMP is an iterative greedy algorithm. For each step, it finds out the column of ***D*** which is the most correlated with the current residuals. This column is then added into the set of selected columns. After the OMP algorithm returns the column set, one can use the least squares (LS) method to further estimate the nonzero values of ***g***. Note that the elements of ***g*** are binary by definition. Such property should be incorporated into the OMP algorithm. Once the column set is obtained, the coordinates of “1” components are also determined. Therefore, we do not need to deal with the LS process before updating the residuals.

The proposed SIF method for recovering ***g*** can be implemented in terms of algorithm 1, referred as the SIF-OMP algorithm. Let us use *q* to denote the iteration number. For any subset ***Γ***_*q*_∈{1,2,…,*L*}, we denote by ***D***_*q*_ a submatrix of ***D*** consisting of the columns $\boldsymbol {d}_{\gamma _{q}}$ within *γ*_*q*_∈***Γ***_*q*_. We use ***r***_*q*_ to denote the residual at the *q*th iteration. Based on the above notations, the SIF-OMP algorithm is given as follows.


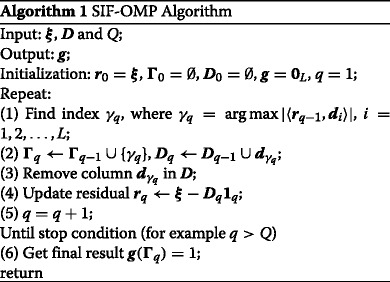


## Simulation results

In this section, we illustrate numerically the performance of the proposed algorithm via various simulations. We consider a similar example given in [15] for ease of comparison. Assume that two BPSK signals with central frequency of 70 MHz and bandwidth of 20% impinge on a ULA with 7 sensors. The code-rate of incident BPSK signals is assumed unknown. First, we show the DOA estimation results of two sources obtained by the MUSIC, MD/MM [14], WCMSR [15], and SIF-OMP, respectively. Within all approaches, the spatial range [−90°,90°] is split into 180 grids with an interval of 1°. The total number of frequency bins is *K*=256. The number of snapshots or segments is *M*=1 and the SNR is set by SNR=20 dB in this example. The parameters *ε* is set to 100 for the MD/MM algorithm. The weight factor is selected by 1 for the WCMSR method.

**Fig. 3:**
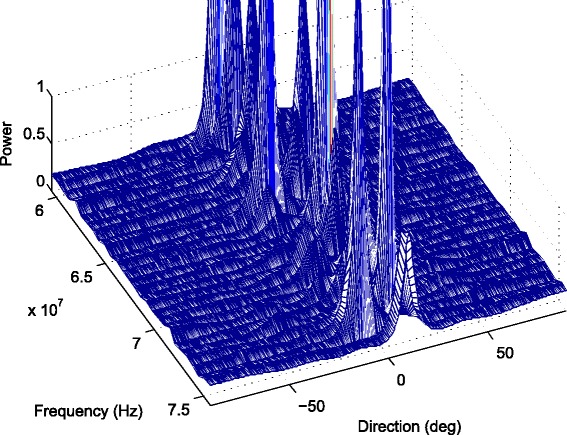
The 3D spectral-spatial spectrum of MUSIC with half-wavelength. The 3D spectral-spatial spectrum of MUSIC with two BPSK signals from −10° and 10° impinging on a ULA interspaced by half-wavelength relative to the highest frequency, SNR: 20 dB, *K*=256 and *M*=1

**Fig. 4:**
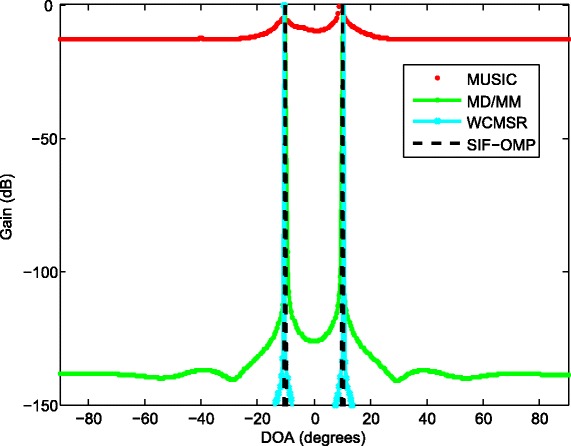
The spatial spectra of two sources with half-wavelength. The spatial spectra of two BPSK signals from −10° and 10° on a ULA interspaced by half-wavelength relative to the highest frequency, SNR: 20 dB, *K*=256 and *M*=1

In first part of simulations, we consider the comparison of the DOA estimates of two sources from the directions of −10° and 10° using the proposed algorithm and previous methods. First, we fix the inter-spacing of the ULA *d* at half-wavelength corresponding to the highest signal frequency. We then enlarge *d* to 100 times to demonstrate the aliasing-free property of the proposed algorithm. In Fig. 3, *d* equals to the half-wavelength with respect to the highest signal frequency and $d=\pi c/\left (\omega _{0}+\frac {B_{f}}{2}\right)$, where *c*=3×10^8^ is the propagation speed of the signal, *ω*_0_=1.4*π*×10^8^ rad/s is the central frequency and *B*_*f*_=0.2*ω*_0_ is the bandwidth. The MUSIC method decomposes the wideband signals into narrowband subbands first and obtains DOA estimates for each frequency bin. Thus, we plot 3D view of spatial spectra with respect to various frequency components and angle grids; see Fig. 3. In Fig. 4, we compare the results of the proposed method with MUSIC, MD/MM, and WCMSR algorithms. The 3D spectrum of the MUSIC method is averaged in angle domain for ease of comparison. All these methods can detect the DOAs successfully for this example.

**Fig. 5:**
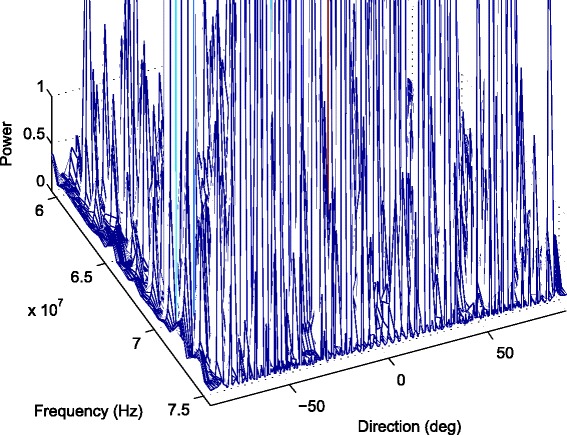
The 3D spectral-spatial spectrum of MUSIC with 100 times half-wavelength. The 3D spectral-spatial spectrum of MUSIC with two BPSK signals from −10° and 10° impinging on a ULA interspaced by 100 times half-wavelength relative to the highest frequency, SNR: 20 dB, *K*=256 and *M*=1

**Fig. 6:**
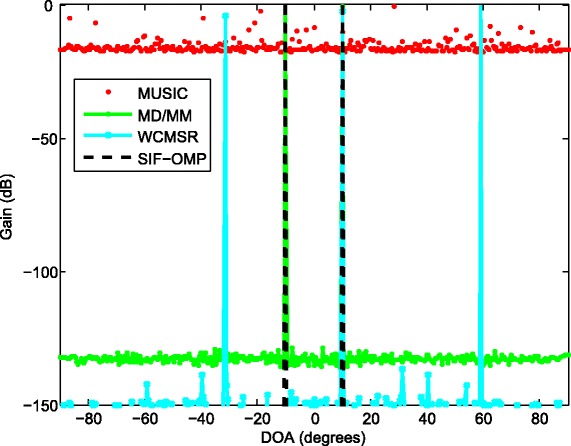
The spatial spectra of two sources with 100 times half-wavelength. The spatial spectra of two BPSK signals from −10° and 10° on a ULA interspaced by 100 times half-wavelength relative to the highest frequency, SNR: 20 dB, *K*=256 and *M*=1

When the inter-spacing of the ULA is expanded 100 times, the phenomenon of spatial aliasing appears for MUSIC and WCMSR algorithms, yet the MD/MM and SIF-OMP methods do not suffer spatial aliasing problem; see Figs. 5 and 6. Figure 5 plots the 3D spectrum of the MUSIC method. The WCMSR also has the spatial ambiguity problem because it works in time domain. Both MD/MM and SIF-OMP algorithms can overcome the spatial ambiguity for this example, and they detect the spectra correctly. With the interval of two adjacent frequencies *Δ* *ω*=2*π*, the spatial nonambiguity of the proposed algorithm is guaranteed if $d<\frac {c}{2}$ according to Theorem 1 in [14].

**Fig. 7:**
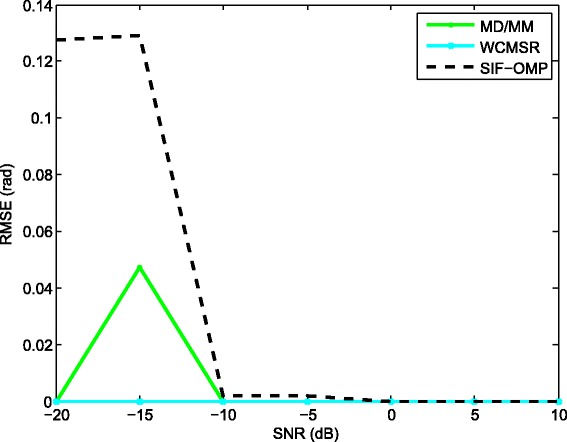
RMSE performance against various SNR (half-wavelength). RMSE performance against various SNR with half-wavelength relative to the highest frequency, *K*=256 and *M*=1

**Fig. 8:**
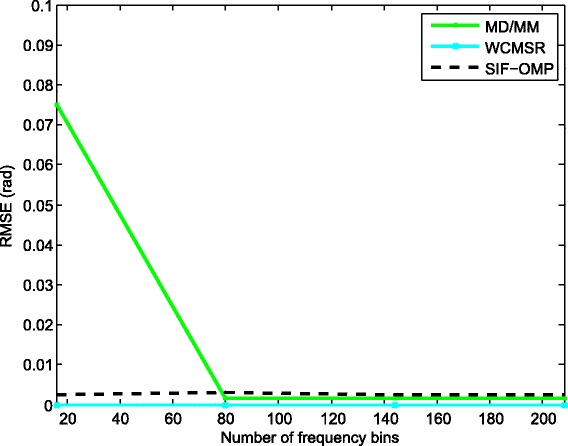
RMSE performance against the number of frequency bins (half-wavelength). RMSE performance against the number of frequency bins with half-wavelength relative to the highest frequency, SNR: 0 dB and *M*=1

**Fig. 9:**
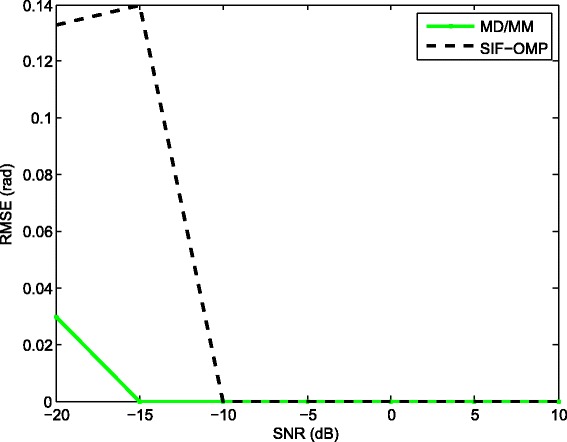
RMSE performance against various SNR (100 times half-wavelength). RMSE performance against various SNR with 100 times half-wavelength relative to the highest frequency, *K*=256 and *M*=1

**Fig. 10:**
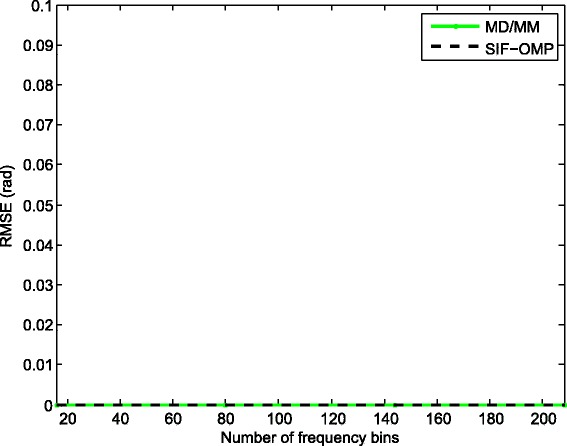
RMSE performance against the number of frequency bins (100 times half-wavelength). RMSE performance against the number of frequency bins with 100 times half-wavelength relative to the highest frequency, SNR: 0 dB and *M*=1

We compare the root mean-squared errors (RMSEs) of the proposed approach with MD/MM and WCMSR algorithms. Please refer to Figs. 7, 8, 9, and 10. The RMSE is defined as follows:

27 $$ \text{RMSE} = \sqrt{E\left(\sum_{q=1}^{Q}\left(\boldsymbol{\hat{g}}_{i_{q}}-\boldsymbol{g}_{i_{q}}\right)^{2}\right)}  $$

where *E*{·} represents the expectation operation. First, we fix the inter-spacing of the ULA at half-wavelength with respect to the highest frequency. The RMSEs of the three methods are summarized in Fig. 7. All methods have satisfactory performance for high SNR. However, the performance of the SIF-OMP method gets worse at low SNR region due to the additional errors. We also examine the RMSEs of the three methods against the number of utilized frequencies when the SNR is fixed at 0 dB; see Fig. 8. Note that the RMSE of the SIF-OMP algorithm does not reduce as the number of frequencies increases. That is because the total value of errors can not be decreased with the increase of frequency bins. We then enlarge the inter-spacing of the ULA to 100 times half-wavelength with respect to the highest frequency and keep the other settings unchanged. Figure 9 plots the RMSEs of the MD/MM and SIF-OMP algorithms with respect to various SNR. Again, we observe that the additional errors affect the performance of the SIF-OMP method for low SNR. Figure 10 shows the RMSEs of the MD/MM and SIF-OMP algorithms against the number of frequency bins when SNR equals 0 dB. These two algorithms both have good performance.

**Fig. 11:**
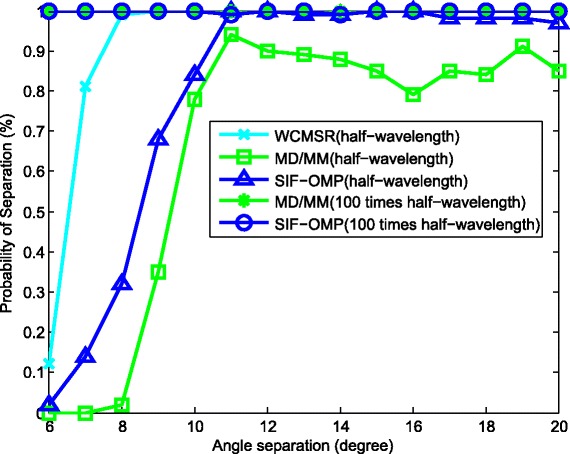
Separation probabilities against various angular separation. Separation probabilities against various angular separation, SNR = 0 dB and K = 256

**Fig. 12:**
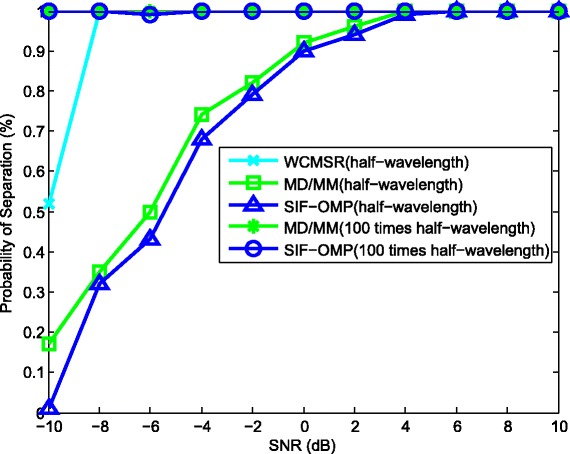
Separation probabilities against various SNR. Separation probabilities against various SNR, source directions: −10°, 0° and *K*=256

**Fig. 13:**
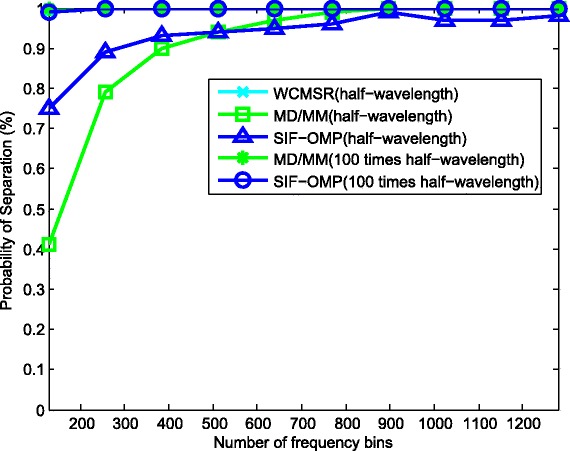
Separation probabilities against the number of frequency bins. Separation probabilities against the number of frequency bins, source directions: −10°, 0° and SNR = 0 dB

We then derive the separation probabilities of those three algorithms in Figs. 11, 12, and 13. A successful separation is defined similarly with [15] when two conditions are satisfied. (1) The amplitude of the highest pseudo-peak is lower than half of that of the lowest signal peak and (2) the bias of the DOA estimates is less than 3°. In the first group of examples, the first source signal is fixed at −10°, and the second one varies from −4° to 10°. For each angle separation, the experiments are done by 100 trials. Figure 11 illustrates the separation probabilities of MD/MM, WCMSR, and SIF-OMP at different angle separations. We observe from Fig. 11 that WCMSR gets better results than MD/MM and SIF-OMP when *d* equals to half-wavelength relative to the highest frequency, whereas the MD/MM and SIF-OMP algorithms can separate two BPSK signals with almost 100% probability when *d* is expanded to 100 times half-wavelength relative to the highest frequency. We then fix the directions of two signals at −10° and 0° and compare the separation probabilities with respect to various SNRs and different number of frequency bins. First, *K* is fixed at 256 and SNR varies from −10 to 10 dB. The separation probabilities of MD/MM, WCMSR, and SIF-OMP are drawn in Fig. 12. Again, we can conclude from Fig. 12 that it is more potential to obtain 100% separation probability when the inter-spacing of the ULA is much larger than half-wavelength. If the SNR is fixed at 0 dB and the number of frequency bins varies from 128 to 1280, their probabilities of separation are plotted in Fig. 13. The angular separation performance of SIF-OMP can be improved when the number of utilized frequencies increases.

## Conclusions

In this paper, we present a new aliasing-free SIF algorithm to solve the wideband DOA estimation problem. The SIF algorithm utilizes all frequency bin information to recover a SIV at the expense of introducing an additional system error. By doing this, we reformulate the MD/MM problem as a single measurement vector recovery problem and therefore reduce the unknown variables greatly. We show that the additional system error can be neglected under certain conditions. After using the binary property of the SIV, we develop a SIF-OMP algorithm to estimate the SIV. We compare the performances of the SIF-OMP algorithm with other well-known methods by analyzing the performance of wideband DOA estimation. The numerical simulations demonstrate the performance of the proposed algorithm.

## Appendices

### Proof of Theorem 1

The constrained optimization problems (13) and (26) can be converted to the following unconstrained optimization problems respectively:

28 $$\begin{array}{*{20}l} & \arg\underset{\boldsymbol{g}}{\min} \left\{\left\|\boldsymbol{\xi}- \boldsymbol{D}\boldsymbol{g}\right\|^{2}+ \lambda\|\boldsymbol{g}\|_{0}\right\}, \end{array} $$

29 $$\begin{array}{*{20}l} & \arg\underset{\boldsymbol{g}}{\min} \left\{\left\|\boldsymbol{Y}-\boldsymbol{X}(\boldsymbol{g})\right\|^{2} + \lambda\|\boldsymbol{g}\|_{0}\right\}, \end{array} $$

where *λ* is the regularization parameter. We need to prove

30 $$ \begin{aligned} &\arg\underset{\boldsymbol{g}}{\min} \left\{\left\|\boldsymbol{\xi}- \boldsymbol{D}\boldsymbol{g}\right\|^{2}+ \lambda\|\boldsymbol{g}\|_{0}\right\}\\ &\quad\equiv \arg\underset{\boldsymbol{g}}{\min} \left\{\left\|\boldsymbol{Y}-\boldsymbol{X}(\boldsymbol{g})\right\|^{2} + \lambda\|\boldsymbol{g}\|_{0}\right\}. \end{aligned}  $$

Note from the expression of ***X***(***g***) in (12), we can obtain

31 $$\begin{array}{*{20}l} &\arg\underset{\boldsymbol{g}}{\min}\left\{\left\|\boldsymbol{Y}- \boldsymbol{X}(\boldsymbol{g})\right\|^{2} + \lambda\|\boldsymbol{g}\|_{0}\right\}\\ &\quad= \arg\underset{\boldsymbol{g}}{\min}\left\{\sum_{k=1}^{K}\sum_{m=1}^{M}\left\| \boldsymbol{y}_{k,m}- \boldsymbol{\Psi}_{k,m}\boldsymbol{g} \right\|^{2} + \lambda\|\boldsymbol{g}\|_{0}\right\} \end{array} $$

As for the optimization problem of (26), we have the following results:

32 $$ {\begin{aligned} &\arg\underset{\boldsymbol{g}}{\min}\left\{\left\|\boldsymbol{\xi}- \boldsymbol{D}\boldsymbol{g}\right\|^{2} +\lambda\|\boldsymbol{g}\|_{0}\right\} \\ & \qquad = \arg\underset{\boldsymbol{g}}{\min}\left\{\boldsymbol{g}^{T}\boldsymbol{D}^{H}\boldsymbol{D}\boldsymbol{g}-2\boldsymbol{g}^{T}\boldsymbol{D}^{H}\boldsymbol{\xi} +\boldsymbol{\xi}^{H}\boldsymbol{\xi} +\lambda\|\boldsymbol{g}\|_{0}\right\} \\ & \qquad = \arg\underset{\boldsymbol{g}}{\min}\left\{\boldsymbol{g}^{T}\boldsymbol\Phi\boldsymbol{g}-2\boldsymbol{g}^{T}\boldsymbol{h} +\sum_{k=1}^{K}\sum_{m=1}^{M}\boldsymbol{y}_{k,m}^{H}\boldsymbol{y}_{k,m} +\lambda\|\boldsymbol{g}\|_{0}\right\} \\ & \qquad = \arg\underset{\boldsymbol{g}}{\min}\left\{\sum_{k=1}^{K}\sum_{m=1}^{M}\left\| \boldsymbol{y}_{k,m} - \boldsymbol{\Psi}_{k,m}\boldsymbol{g} \right\|^{2} +\lambda\|\boldsymbol{g}\|_{0} \right\} \end{aligned}}  $$
